# Incentivizing performance in health care: a rapid review, typology and qualitative study of unintended consequences

**DOI:** 10.1186/s12913-022-08032-z

**Published:** 2022-05-23

**Authors:** Xinyu Li, Jenna M. Evans

**Affiliations:** 1 Faculty of Health Sciences, McMaster University, Hamilton, Canada; 2 DeGroote School of Business, McMaster University, 1280 Main Street West, Hamilton, ON L8S4M4 Canada

**Keywords:** Performance management, Performance measurement, Quality indicators, Unintended consequences, Health systems

## Abstract

**Background:**

Health systems are increasingly implementing policy-driven programs to incentivize performance using contracts, scorecards, rankings, rewards, and penalties. Studies of these “Performance Management” (PM) programs have identified unintended negative consequences. However, no single comprehensive typology of the negative and positive unintended consequences of PM in healthcare exists and most studies of unintended consequences were conducted in England or the United States. The aims of this study were: (1) To develop a comprehensive typology of unintended consequences of PM in healthcare, and (2) To describe multiple stakeholder perspectives of the unintended consequences of PM in cancer and renal care in Ontario, Canada.

**Methods:**

We conducted a rapid review of unintended consequences of PM in healthcare (*n* = 41 papers) to develop a typology of unintended consequences. We then conducted a secondary analysis of data from a qualitative study involving semi-structured interviews with 147 participants involved with or impacted by a PM system used to oversee 40 care delivery networks in Ontario, Canada. Participants included administrators and clinical leads from the networks and the government agency managing the PM system. We undertook a hybrid inductive and deductive coding approach using the typology we developed from the rapid review.

**Results:**

We present a comprehensive typology of 48 negative and positive unintended consequences of PM in healthcare, including five novel unintended consequences not previously identified or well-described in the literature. The typology is organized into two broad categories: unintended consequences on (1) organizations and providers and on (2) patients and patient care. The most common unintended consequences of PM identified in the literature were measure fixation, tunnel vision, and misrepresentation or gaming, while those most prominent in the qualitative data were administrative burden, insensitivity, reduced morale, and systemic dysfunction. We also found that unintended consequences of PM are often mutually reinforcing.

**Conclusions:**

Our comprehensive typology provides a common language for discourse on unintended consequences and supports systematic, comparable analyses of unintended consequences across PM regimes and healthcare systems. Healthcare policymakers and managers can use the results of this study to inform the (re-)design and implementation of evidence-informed PM programs.

## Introduction

Health systems are increasingly implementing policy-driven programs to incentivize performance in healthcare organizations and networks using contracts, targets, scorecards, rankings, rewards, and sanctions. These “Performance Management” (PM) programs provide performance feedback and establish accountability for performance outcomes with the aim of influencing behavior and results [1]. Studies of PM in healthcare demonstrate that in addition to contributing to improvement PM can generate unintended consequences for organizations, providers, and patients [2–6]. Much of the literature on unintended consequences stems from the English National Health Service (NHS) where a centralized “command-and-control” approach to PM has been criticized for contributing to measure fixation, gaming, and reduced staff morale [5, 7–10]. Increasingly, studies from the United States also report unintended consequences of PM, citing many of the same issues identified in England in addition to concerns about the role of PM in widening racial and socioeconomic disparities [11–16].

Typologies of the unintended consequences of PM in healthcare [2, 5] and in public management [17, 18] exist. However, there is no single comprehensive typology of the negative *and* positive unintended consequences of PM in healthcare. Furthermore, few studies of the unintended consequences of PM have been conducted outside of England and the United States, which limits our understanding of unintended consequences across PM regimes and healthcare systems. The aims of this study were twofold: (a) To develop a comprehensive typology of unintended consequences of PM in healthcare, and (b) To describe multiple stakeholder perspectives of the unintended consequences of a PM system in Ontario, Canada.

## Methods

### Rapid review

We conducted a rapid review of the literature on unintended consequences of PM in healthcare [19]. A rapid review was appropriate because our intent was to develop a typology, not to synthesize, assess, and critique the body of evidence on unintended consequences of PM.

**Fig. 1:**
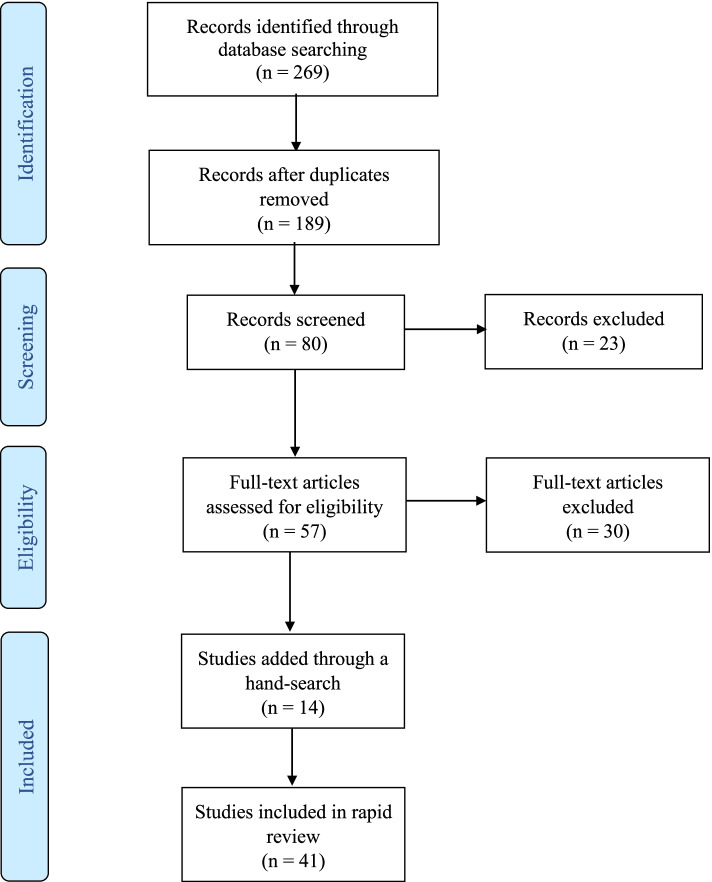
PRISMA Flow Diagram for Rapid Review on Unintended Consequences of Performance Management in Healthcare

A search of the academic literature was conducted in Fall 2020 and updated in Fall 2021 using the electronic databases PubMed and Web of Science (All Databases). The following search string was used for both databases: ((“healthcare” OR “health care” OR “health system”) AND (“performance management” OR “performance incentives” OR “performance feedback” OR “performance measurement” OR “quality indicators” OR “quality measure*”) AND (“unintended consequences” OR “unintended effects” OR “unintended responses” OR “unintended negative consequences” OR “perverse effects” OR “dysfunctional consequences” OR “unintended positive consequences” OR “unintended benefits”). To be included, papers had to be peer-reviewed academic papers (grey literature, conference proceedings, policy statements, and clinical guidelines were excluded), published in English, and involve a conceptual/theoretical discussion or empirical evidence on unintended consequences of PM in healthcare. No limits were placed on year of publication. Two authors independently screened papers for inclusion and met to discuss and finalize screening decisions. A hand search of the reference lists of included papers was also conducted to identify relevant papers. Ultimately, 41 papers were included in the review (Fig. 1). Excluded papers – while relevant to performance measurement and management – had minimal discussion of unintended consequences.

The following information was extracted from each paper to facilitate synthesis: Reference; Study Purpose; Methods; PM Intervention(s) Studied; Definitions, Theories, or Frameworks of Unintended Consequences; Types of Unintended Consequences; Results; and Recommendations for Preventing/Managing Unintended Consequences. Two authors independently extracted data for three papers and met to discuss and finalize the extraction approach. Extracted data were compared and synthesized to create a typology of all unintended consequences identified in the literature. This process was undertaken collaboratively by the authors and involved merging similar categories and minor modifications to wording for accuracy and clarity.

### Qualitative study setting

The province of Ontario is in Central Canada and has a population of 14.5 million. Cancer Care Ontario (CCO), which houses the Ontario Renal Network (ORN), is a government agency with a mandate to fund, oversee, and improve cancer and renal care. Delivery of cancer and renal care is organized by geographic region through 13 regional cancer networks and 27 regional renal networks that together cover the full geography of the province.

As an oversight body, CCO uses a robust PM system to monitor and improve performance of cancer networks (since 2005) and renal networks (since 2013). CCO’s PM system consists of the following components: funding contracts outlining performance expectations/deliverables with funding at risk of withdrawal for non-compliance; a regional scorecard with indicators, targets, and network rankings; access to performance data through electronic platforms; quarterly performance review reports and meetings; annual performance recognition certificates; an escalation process for poor or declining performance; and public reporting of performance on select indicators [20]. CCO’s PM system is described in more detail in Table 1. Available evidence suggests that CCO and its PM system are high-performing and internationally renowned [20–24]. Hagens et al. [20] found that 89% of cancer care indicators prioritized by CCO over the past 15 years demonstrated sustained improvement over time. Furthermore, Ontario’s performance on most cancer mortality and cancer survival indicators exceeds the Canadian and OECD averages [22]. Finally, CCO has been described in media outlets as an “internationally leading agency” [21], “the envy of the world” [24], and “the pinnacle of success in establishing standards, tracking and publicly reporting outcomes, multi-year planning, developing information systems, consulting patients, and advising government and practitioners” [24].

**Table 1:** Cancer Care Ontario (CCO) Performance Management Interventions Organized by Primary Function

PM Intervention	Description
**Guiding**	
Network administrative and clinical leadership model	Administrative leaders are embedded in host hospitals and are employees of, and therefore accountable to, both their host hospital and CCO. Each clinical program within CCO has a ‘clinical lead’ counterpart within each network. Clinical leads are paid by CCO for one day per week.
Funding contracts	Specify expectations for patient volumes, data submission, and implementation of initiatives. Some funds may be withdrawn for non-compliance with these performance expectations/deliverables.
**Monitoring**	
Scorecard	Generated quarterly to grade performance for each network (green, yellow, red) relative to each indicator and its target, and to rank performance of each network relative to others (global ranking in cancer for overall performance versus ranking per indicator in renal)
Annual Monitoring Report	Generated annually to monitor indicators retired from the scorecard (cancer only).
Web-based access to performance data	Secure, web-based database and analytic tools offer historic, current, and projected data on indicators; updated monthly and allows users to generate reports on specific queries.
Public reporting	The Cancer Quality Council of Ontario generates the Cancer System Quality Index, which is a web-based public reporting tool on cancer system performance across 30 indicators. Select renal indicators are also reported on the Ontario Renal Network website.
**Improving**	
Quarterly performance review reports	Issued quarterly in preparation for quarterly meetings (below) and includes scorecard indicators as well as additional indicators; if performance is below target or declining for any given indicator, requires commentary on contributing factors and improvement plans; space also provided to summarize successes
Quarterly performance review meetings	Held between CCO leaders and network leaders following each quarter. Occurs via video-conference in cancer system and tele-conference in renal system. Performance results for the past quarter are discussed as well as data quality or reporting problems, success stories, challenges, and plans for improvement.
Recognition certificates	Issued annually for each indicator for networks that met target, were the top performer, and/or were the most improved.
Escalation process for poor or declining performance	Commences with an informal conversation with network leadership regarding performance, and may progress to a formal letter with template to complete a required improvement action plan and, in rare cases, to withdrawing of funds associated with requirement, if relevant.

In 2019, after the completion of data collection, CCO was incorporated into a new agency, Ontario Health. CCO’s programs and services remain unchanged and they continue to be referred to as “CCO” within Ontario Health.

### Secondary analysis of qualitative interviews

We conducted a secondary analysis of semi-structured interview data from a qualitative constructivist study [25] on CCO’s PM system. Administrative and clinical representatives working at CCO and in the regional networks who were involved with or impacted by PM were invited via e-mail to participate in individual or group interviews using purposeful and snowball sampling. Participants provided informed consent verbally at the start of each interview. Participants were asked open-ended questions regarding CCO’s role and the strengths and weaknesses of CCO’s PM system using a pre-tested semi-structured interview guide. The one-hour interviews took place in-person or on the phone, depending on geographic location, with no non-participants present, and were digitally recorded and transcribed verbatim. All interviews were conducted between 2017 and 2018 by the second author, who holds a PhD, specializes in qualitative and mixed methods research, and worked with CCO as a Staff Scientist at the time of data collection. She was hired, in part, to establish a program of research on PM. Six participants from CCO had a prior relationship with the interviewer through other projects. Data saturation was reached with approximately half of the final sample of participants, but interviews continued with the aim of achieving broad stakeholder engagement and input into PM, which is a core function of CCO. Refusals to participate were minimal and related to time and scheduling challenges. No participants dropped out.

Unintended negative consequences were explored through one interview question in the primary study (“Tell me about the unintended negative consequences of PM, if any”), but it was not a central part of the primary analysis. That data were originally coded by the second author as “unintended consequences” using NVivo software. The first author conducted a more detailed coding of that sub-set of the data in NVivo. The first author coded the data deductively, classifying the unintended consequences according to the typology developed from the rapid review. Upon completion, the second author reviewed the node reports for accuracy and coding disagreements were resolved through discussion and rectified in NVivo. The authors discussed and agreed on the addition of new inductive codes, where necessary, and updated the typology accordingly. Emerging results were shared with participants via email and/or presentations where they were encouraged to ask questions, offer feedback, and share their interpretations of the data.

In the participant quotes provided below, we use the notation “P” for individual interviews and “G” for group interviews. In group interviews, it was not always possible to identify the role of the speaker (administrative versus clinical) or the clinical area they represent (cancer versus renal); therefore, some quotes indicate role and clinical area while others do not.

## Results

We identified 48 unintended consequences of PM in healthcare, which we organized based on who was impacted – providers and organizations or patients and patient care – and on the nature of impact – negative or positive. Table 1 presents the final typology incorporating both the results of the rapid review and qualitative study.

### Rapid review

The review included 41 papers published between 2002 and 2020 consisting primarily of qualitative studies, literature reviews, and discussion papers. Although several typologies of unintended consequences were available in the literature, none were comprehensive [2, 5, 18].

Most of the unintended consequences described in the literature focused on the impact of PM on providers and organizations. We divided these unintended consequences into six subcategories, retaining much of the language and content of the typology proposed by Mannion and Braithwaite [5]: (a) increased work, (b) poor design or use of performance data, (c) breaches of trust and increased work environment toxicity, (d) exacerbation of inequalities, (e) politicization of performance management and (f) positive unintended consequences. The most common unintended negative consequences on providers and organizations were ‘measure fixation’, ‘tunnel vision’, and ‘misrepresentation’ and ‘gaming’ (Table 2). The most common positive unintended consequence for providers and organizations was ‘improved morale’ due to the sense of pride that comes with high performance (Table 2).

**Table 2:** Typology of Unintended Consequences of Performance Management in Healthcare

Type	Description	References	In Our Data?
**Unintended Consequences on Providers and Organizations**			
***I. Increased Work***			
a. Increased administrative burden	Excessive time spent on administrative tasks (e.g., documentation, data collection and submission, justifying deviation from clinical reminders when not clinically relevant)	[6, 13–16, 26–31]	✓✓
***II. Poor Design or Use of Performance Data***			
a. Tunnel vision	Emphasis is placed on dimensions of performance that are measured or incentivized, while other unmeasured but important aspects are overlooked	[2, 3, 5, 10, 13, 14, 28, 30–39]	✓✓
b. Measure fixation	Emphasis is placed on meeting the performance target rather than the associated objective	[2, 3, 5, 10, 26, 28, 32, 33, 38, 40–42]	✓✓
c. Suboptimization	Focusing on one component of a total and making changes intended to improve that one component and ignoring the effects on other components (e.g., pursuit of narrow local objectives at the expense of broader organizational or system objectives)	[2, 10, 33, 41]	✓
d. Myopia	Excessive concentration on short-term targets without consideration for long-term consequences	[2, 5, 10, 33, 41, 43]	✓
e. Quantification privileging	Fixation on data that can be quantified causing qualitative aspects of healthcare to be missed	[5, 34, 44]	✓
f. Anachronism	Lag effect between data capture and data usage causes data to not help solve current problems	[5]	✓
g. Insensitivity	Assessment does not capture overall complexity of health performance, causing the wrong providers, units, or organizations to be penalised or rewarded (e.g., contextual factors not considered, risk adjustment not performed, good performance results in a disadvantage such as improved efficiency and cost savings resulting in a lower budget the following year)	[3, 5, 6, 10, 31, 37, 39, 45, 46]	✓✓
h. Misinterpretation	Incorrect inferences made about raw performance due to a lack of understanding of the measure and its underlying methodology or to a failure to account for the full range of possible influences on performance	[2, 5, 47]	✓
i. Complacency	Reduced ambition to improve caused by the perception that performance is satisfactory	[5, 41, 48]	✓
j. Fossilization	PM system excessively rigid to the point of organizational paralysis and reduced innovation (e.g., choosing not to adopt new technology or procedure so that current performance is maintained)	[2, 5, 10, 33, 42, 49]	✓
k. Systemic dysfunction	Performance priorities, indicators, measurement methodologies, interpretations of data, and/or resulting actions are misaligned or contradictory across programs and hierarchical levels within an organization or between PM schemes that co-exist in the broader healthcare system	[10]	✓✓
l. Resource waste	Time and money are spent on PM without achieving its underlying objectives; time and money spent on unnecessary care	[31, 50]	✓
***III. Breaches of Trust & Increased Toxicity of the Work Environment***			
a. Misrepresentation	Deliberate manipulation of data to appear a better performer (e.g., creative accounting, fraud, upcoding)	[2, 3, 5, 8, 33, 35, 39, 42, 51–55]	✓
b. Gaming	Deliberate manipulation of behavior to appear a better performer (e.g., cherry-picking patients, stopping the clock for wait time indicators)	[2, 3, 5, 8, 33, 35, 36, 39–42, 49, 51, 52, 54, 56]	✓
c. Bullying	Pressure for performance improvement involves shaming, intimidating, or coercing staff; PM system seen as punitive and oppressive	[5, 9, 32]	
d. Loss of professional ethos/morality	PM causes provider motivation to shift from providing the best care to providing incentivized care, thereby undermining providers’ intrinsic motivation	[12, 13, 51, 52]	
e. Reduced learning and psychological safety	A work environment focused on blame emerges and generates distrust and fear that inhibits problem-solving, learning, and innovation	[7, 12, 15, 32, 40, 41]	
f. Reduced autonomy, agency and/or self-regulation	PM reduces individual, organizational, or network autonomy, agency, and ability to self-regulate due to the PM system itself and/or due to how PM was implemented (i.e., imposed on providers, rather than designed and undertaken with or by them)	[6, 9, 14, 32, 34, 56, 57]	✓
g. Reduced morale	Loss of belief and confidence in their organization’s mission, goals, or work or loss of belief and confidence in PM tools and processes	[5, 9, 10, 26, 27, 32, 40, 41, 52]	✓✓
h. Team and inter-professional conflict	Reduced cooperation and increased tension between teams and professional groups due to PM	[26, 27, 32, 36]	
i. Increased perceived injustice (due to social comparisons)	Feelings of competition, resentment, and frustration between those individuals and groups who are affected by PM and those who are not (e.g., those not affected by PM do not receive the same attention and/or resources; those affected by PM operate under more scrutiny and pressure)	N/A	✓
j. Toxic ambition	Constant pressure to improve even when performance meets or exceeds the target	N/A	✓
***IV. Exacerbation of Inequities***			
a. Increased resource gap	Providers that treat poorer or underserved patients may have less resources to invest in improvement. As a result, they perform worse and then either do not benefit from incentives or experience penalties that further exacerbate existing resource gaps	[5, 11, 32, 45, 46, 48, 49, 52–60]	✓
b. Reduced ability to recruit necessary staff	Staff are attracted to highly rated organizations compared to lower rated organizations thereby making it more difficult for lower rated organizations to recruit staff and improve performance	[5, 35]	
c. Overcompensation	Incentive payments made are higher than required to meet performance targets, thereby reducing resources for other important types or aspects of care	[5]	
***V. Politicization of Performance Management***			
a. Political grandstanding	PM is driven by interests of governments, political parties, the media and other stakeholders	[5]	
b. Political diversions	PM is used as a distraction by governments under pressure	[5]	
***VI. Positive Unintended Consequences***			
c. Improved morale	Feeling of recognition and increased confidence and pride in individual or organizational performance	[13, 16, 40, 61]	✓
d. Motivated learning and development	PM spurs further education and training to support improvement	[13, 27]	✓
e. New relationships and collaborative problem-solving	Professionals, organizations, or networks come together in new and inventive ways to cope with PM	[9, 26, 36]	✓✓
f. Improved capacity planning	Information collected through PM allows for better internal planning and external applications	N/A	✓
**Unintended Consequences on Patients and Patient Care**			
***I. Inappropriate or Sub-Optimal Care***			
a. Clinical decisions driven by PM (rather than by evidence and clinical judgment)	PM generates pressure to diagnose and treat patients in particular ways, resulting in under-treatment, over-treatment, and/or harm to patient	[9, 12, 13, 15, 27, 28, 38, 40, 49, 50, 56, 57, 62–64]	✓
b. Improved documentation without improved care	Providers document care provided more effectively, but the care itself is not improved	[6, 13, 39, 49, 54, 55]	✓
c. Less continuity of care	When PM incentivizes approaches to care that result in patients interacting with multiple providers rather than or in addition to their primary provider(s) (e.g., incentives for same-day appointments and after-hours care)	[4, 40]	
***II. Reduction in Patient-Centered Care***			
a. Compromised patient education and treatment choice	Providers promote incentivized treatments over non-incentivized treatments to patients or fail to obtain informed consent before conducting a test or procedure	[6, 15, 38, 40]	✓
b. Compromised patient autonomy	Providers exert pressure on patients who refuse incentivized care	[6, 15, 40]	✓
c. Compromised patient convenience	Causing inconveniences for patients for purposes relating to PM (e.g., requiring an additional appointment that would otherwise not be deemed necessary or bringing up a topic like end-of-life care at an inappropriate time and place)	[3, 14]	✓
d. Compromised patient engagement	Reduction in patient engagement as a result of changes in treatments driven by PM	[65]	
e. Disregard for the patient voice	Providers give less attention and priority to patient concerns and preferences compared to PM-related aspects of care	[6, 14, 15, 38, 40, 44, 54, 63]	✓
f. Erosion of trust in care	Patients lose confidence in their healthcare providers after a poor performance assessment or after experiencing or witnessing manipulation driven by PM	[4, 5, 34, 35, 66]	
***II. Exacerbation of Inequities***			
a. Increased inequity in access to high quality care	Providers avoid high risk or socially challenging patient sub-groups or choose patients who can maximize positive measurement (i.e., cherry-picking) When financial incentives for high performance are re-invested in improved services, then patients of high-performing services benefit to a greater degree than patients of under-performing services	[5, 11, 37, 43, 46, 54, 56, 58, 63, 67]	✓
b. Increased healthcare disparities	Increased health care disparities in the population based on sex, race, ethnicity, language, or economic status due to (1) differences in access to high quality care or (2) improvements spurred by PM are more useful to mainstream patients	[11, 33, 37, 40, 48, 51, 58, 62, 63, 67]	✓
***IV. Positive Unintended Consequences***			
a. Beneficial spillover effects	PM contributes to improved performance in other clinical areas that are not performance managed	[4, 13, 36, 59, 68]	
b. Increased patient knowledge	PM increased patient education efforts	[61]	
c. Increased patient motivation and engagement with care	Patients more involved and compliant with recommended care due to increased education and time spent	[61]	
d. Increased patient satisfaction with care	PM promoted more comprehensive care (e.g., addressing multiple issues per visit, including preventive care)	[61]	
e. Enhanced patient-provider communication and relationships	PM increased patient-provider communication, resulting in positive psychological feelings among patients regarding their providers and their care	[4, 61]	

The second major category of unintended consequences were those that affect patients and patient care, which we divided into four subcategories: (a) inappropriate or sub-optimal care, (b) reduction in patient-centered care, (c) exacerbation of inequalities, and (d) positive unintended consequences. The most common unintended negative consequences in this category were ‘clinical decisions driven by PM’ rather than by evidence and clinical judgment and ‘increased inequality in access’ or ‘increased healthcare disparities’ (Table 2). Few studies explicitly examined positive unintended consequences on patients and patient care [61]. However, ‘beneficial spillover effects’ were identified in which PM contributed to improved performance in other non-incentivized clinical areas for the target population (Table 2).

Several strategies were proposed to mitigate the unintended negative consequences of PM. The most common recommendation was greater collaboration among developers of PM systems, those responsible for implementation, and providers and patients affected by PM – with the aim of maximizing alignment of PM systems with the local context, provider goals and values, and patient priorities [5–7, 9, 12, 14–16, 32–34, 40, 44]. This collaboration should be ongoing, occurring not just during the design phases, but also over time, allowing for feedback, reflexive review, and modifications to PM [2, 5, 10, 12, 14, 51]. Many recommendations also focused on the characteristics of effective indicators (e.g., evidence-based, balanced) and transparency of the indicator selection and measurement processes [5, 35, 43, 47, 50].

### Unintended consequences of performance management in cancer and renal care in Ontario

We analyzed interview data from 147 clinical leads and administrative staff members. Fifty-nine were representatives from CCO (85% in an administrative role and 15% in a clinical role). The remaining 88 participants were from cancer and renal networks (74% in an administrative role and 26% in a clinical role). Twelve of the 14 cancer networks (86%) and 19 of the 27 renal networks (73%) were represented.

Before describing the results of our secondary analysis of unintended consequences of PM, it is important to reiterate that previous research, international health data comparisons, and media reports establish CCO and its PM system as high-performing and internationally leading [20–24]. Our qualitative study supports this conclusion. Participants’ perceptions of CCO’s approach to PM were positive, highlighting province-wide priority-setting, benchmarking, improvement initiatives, and sharing of best practices as strengths, while recognizing opportunities for refinement.

**Fig. 2:**
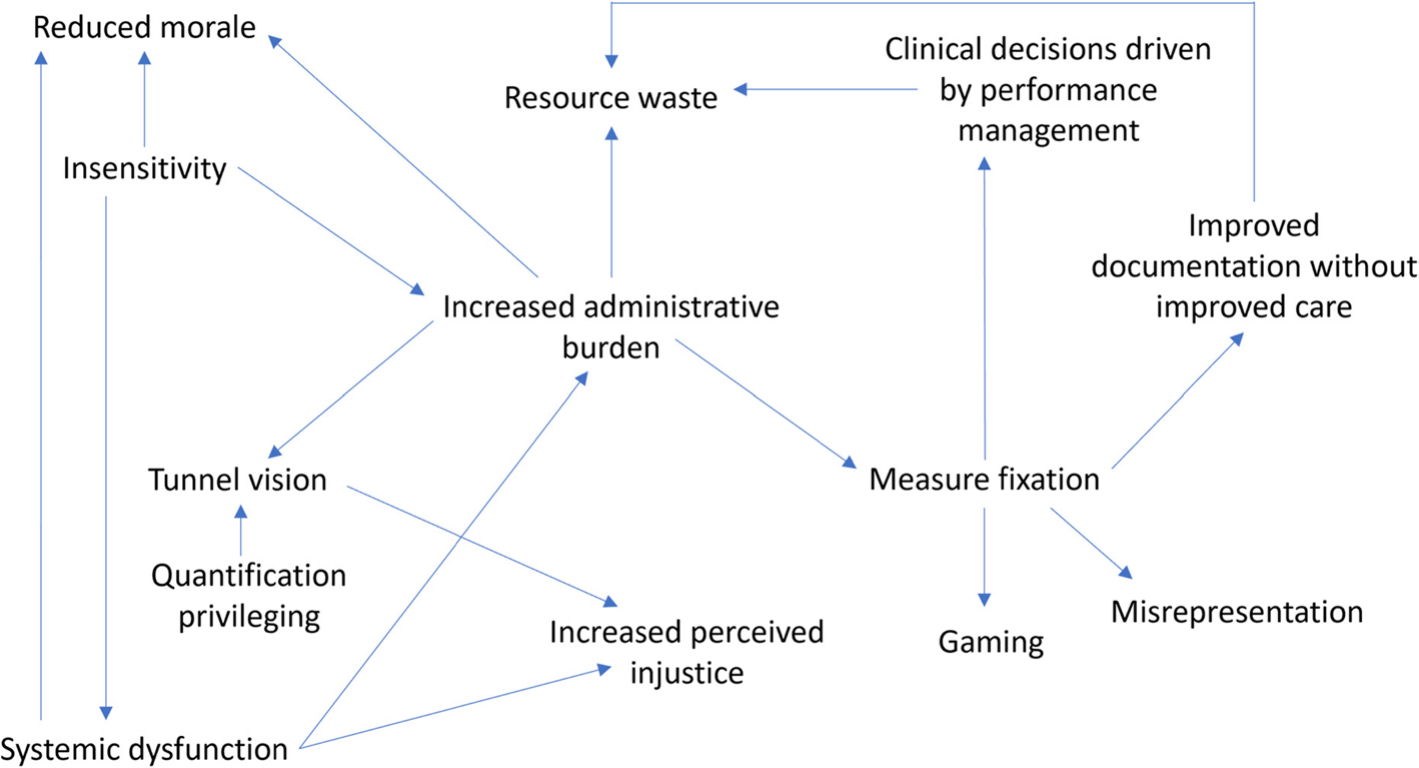
Relationships Among Unintended Consequences Based on Interview Data

We found that most of the unintended consequences identified by participants focused on organizations and providers, not patients and patient care. Even though participants were only asked to reflect on *negative* unintended consequences of PM, we did identify examples of positive unintended consequences. Overall, the most common unintended consequences were ‘increased administrative burden’, ‘insensitivity’, ‘reduced morale’, and ‘systemic dysfunction’. We also identified 5 unintended consequences not captured by previous typologies and rarely identified in existing literature: (a) systemic dysfunction, (b) resource waste, (c) increased perceived injustice, (d) toxic ambition, and (e) improved capacity planning. Below we summarize results for each category of unintended consequences. In Fig. 2, we map how the unintended consequences mutually reinforced one another.

### Negative unintended consequences on providers and organizations

#### Increased work

‘Increased administrative burden’ was the most common unintended consequence of PM reported by participants across all stakeholder groups and among all categories in the typology. Participants’ primary concern was the volume of data that is collected, processed, and reported, as these two participants explained:

*“We probably have more staff tied up in data submissions and data quality work than most of the rest of the hospital. It just seems like a really labour-intensive, very resource-intensive requirement”* (G21, Network, Cancer and Renal)

*“Our resources don’t increase, our staff don’t increase, but the demand on us increases exponentially from one quarter to the next”* (G06, Network, Renal)

The administrative burden described by participants was often linked to two contributing factors. The first factor was the number of required performance indicators, as this CCO representative acknowledged: *“It’s the sheer number of indicators that we try to push forward. At some point, you’re just diluting the capacity that exists within the regions or in the hospitals to make change”* (P71, CCO, Cancer, Administrator). The second contributing factor was the inconsistencies in data systems and PM requirements across the multiple oversight bodies to whom networks are accountable as this participant explained: *“For me, the big thing is 100% the data burden. They forget that we have shared accountabilities. We’re not just accountable to CCO”* (G06, Network, Renal). Many participants described conflicting data requirements between organizations and oversight bodies such as their own hospital, CCO, the Canadian Institute for Health Information, the Local Health Integration Network, Health Quality Ontario, and Accreditation Canada. As such, the administrative burden was spurred in part by the unintended consequence of ‘systemic dysfunction’, described further below.

Workload concerns were also closely related to the unintended consequence of ‘resource waste’ as many participants expressed concern about whether the resource investment in PM was meaningful and contributing to improvement. There were also concerns that the workload associated with PM results in over-attention to PM, potentially reinforcing the unintended consequences of ‘tunnel vision’ and ‘measure fixation’, described below.

#### Poor design or use of performance data

Regarding the design of PM interventions and use of performance data, the most common unintended consequences identified by participants were ‘insensitivity’, ‘measure fixation’, ‘tunnel vision’, and ‘systemic dysfunction’ (Table 2).

‘Insensitivity’ refers to PM failing to capture the complexity of healthcare delivery and performance, potentially resulting in unfair penalties or rewards. In our data, insensitivity manifested primarily as a perceived a lack of control over performance due to contextual differences between networks and/or the nature of indicators. First, some participants argued that PM neglects regional differences that can impact network performance, such as differences in geography, resources, and patient demographics:

*“There’s a lot of unmeasured confounding in some of these measures. So, we think everybody should be able to get there, but, you know, everything falls within a distribution, and someone’s going to be an outlier, and it’s not necessarily their fault that they’re an outlier”* (P36, Network, Clinical Lead)

Second, some network representatives argued that select indicators reflect service performance beyond their immediate control. CCO representatives acknowledged that some indicators are aimed at stimulating collaboration across programs within and across organizations.

‘Measure fixation’ involves placing emphasis on meeting the performance target rather than the associated objective. Participants shared examples and raised questions regarding the extent to which PM inadvertently distracts attention away from patient care:

*“People are really focused on numbers and really focused on the methodology. They’re not necessarily focused on what’s ultimately best for the patient because they want to look good on the scorecard and rankings”* (G14, CCO, Clinical Leads)

*“Sometimes I feel if we just play the game we could be a perfect performer, but I’m not sure our patients would be any better off…we would just learn how to play the game of being a good performer”* (G22, Network, Cancer)

‘Tunnel vision’ occurs when emphasis is placed on dimensions of performance that are measured or incentivized, while other unmeasured but important aspects are overlooked. Participants described tunnel vision in two ways. In the first set of examples, participants described how indicators tend to focus on select fragments of the patient journey, and on processes like wait times rather than outcomes like patient survival, which one participant described as *“losing the forest for the trees”* (G11, Network, Cancer). Some of these indicator-focused examples were related to the unintended consequence of ‘quantification privileging’. In the second set of examples, participants focused more broadly on the healthcare system, explaining how PM in one part of the healthcare system (in this case, in cancer care) can exacerbate problems in other parts of the system that do not operate under the same PM requirements:

*“Sometimes the cancer program is seen as the have-more program and other disease states, for people that present to hospital, are maybe the have-less. For example, gastrointestinal does not have a provincial body that’s driving performance. Or rheumatology, for example. So, those patients, I’m hearing, are being bumped or less prioritized for surgery and things like that, because they’re not associated with, for example, a surgical wait time metric”* (G18, Network, Cancer)

These system-level examples of ‘tunnel vision’ seemed to contribute to the unintended consequence of ‘increased perceived injustice’, described in the next section.

We also identified examples of or concerns about ‘systemic dysfunction’ and ‘resource waste’. These unintended consequences are not included in previous typologies and are rarely discussed in the literature. ‘Systemic dysfunction’ was a prominent theme with participants describing inconsistent priorities, measurement methodologies, and data interpretations between hierarchical levels within their organization or between oversight bodies in the healthcare system. This systemic dysfunction was described as pre-existing in the healthcare system and exacerbated by PM requirements that reinforce conflicts or misalignments.

*“We’re often collecting the same data slightly differently for all four organisations… The data tends to not take on the same meaning as it should if everybody was measuring it and looking at it together”* (P61, Network)

*“Sometimes your own hospital strategy or direction may be in conflict with what [CCO] is trying to do so you’re trying to always play this balancing game”* (G24, Network, Renal)

‘Systemic dysfunction was viewed as contributing to the unintended consequence of ‘increased administrative burden’, described earlier.

The unintended consequence of ‘resource waste’ was implied in participants’ descriptions of the workload associated with PM and their reflections on the impact of PM on patient care. Participants often wondered about the cost-benefit of PM:

*“Remember, there’s financial cost and opportunity cost for the amount of time we spend on PM”* (G20, Network, Cancer and Renal)

*“It does seem disproportionate in terms of the amount of effort we put into measuring particular indicators that don’t necessarily have a return on patient experience or patient outcome”* (G21, Network, Cancer and Renal)

We identified very few examples of each of the remaining seven unintended consequences in this category: suboptimization, myopia, quantification privileging, anachronism, misinterpretation, complacency, and fossilization.

#### Breaches of trust & increased toxicity of the work environment

Regarding the unintended consequences of PM on the work environment, many participants used language that reflected ‘reduced morale’, most often in terms of a loss of belief and confidence in PM (not in their organization or their work). Negative emotive descriptors like “frustrating’ and ‘embarrassing’ were common among network representatives. However, CCO representatives were more likely to reflect on the impact of PM on morale in networks struggling with performance using stronger terms like ‘discouraging’, ‘depressing’, or ‘demoralizing’. Reduced morale was closely linked with the unintended consequence of ‘insensitivity’, as this quote from a CCO representative demonstrates:

*“They don’t feel a strong motivation to work toward achieving the provincial benchmark, if they feel like they’re never going to be able to reach it anyway”* (G03, CCO, Administrators)

In addition to ‘insensitivity’, ‘reduced morale’ was also often coupled with the unintended consequences of ‘increased administrative burden’ and ‘systemic dysfunction’.

Many participants, particularly CCO representatives, expressed concerns about ‘misrepresentation’ of data and ‘gaming’ of the PM system, primarily in reference to wait time indicators:

*“All of a sudden over 50% of their patients were being seen the same day they were referred, which is amazing, and it doesn’t make sense either…I think because they changed how they define the referral date as whatever was convenient to them. So, I think we have to be careful that we don’t push people so far that they start making up information so that we get off their backs”* (P29, CCO, Cancer, Administrator)

*“We’ve had that in wait times reporting, where certain cases are being reclassified to incorrect buckets to make it appear as though they’re being completed on time”* (P61, CCO, Renal, Administrator)

*“My own hospital is absolutely gaming wait times…I have no doubt that there has been no actual improvement in wait times at this institution”* (G14, CCO, Cancer, Clinical Leads)

Another ‘gaming’ behaviour identified in the data was intentionally prioritizing indicators that were more likely to lead to an increase in overall performance and ranking:

*“We sacrifice some indicators for others. For example, we have a look at all the indicators, and know that we’re not going to be able to make a very big dent on this indicator due to…the challenges of our region. So, then we focus on the indicators that we actually know we can make an impact on because we don’t want to be [ranked] 14th”* (P60, Network)

The unintended consequences of ‘misrepresentation’ and ‘gaming’ were closely related to ‘measure fixation’.

‘Reduced autonomy, agency, and/or self-regulation’ also emerged as a negative unintended consequence of PM. Although we identified one example of this at the individual provider level, most accounts were focused on the network level:

*“[CCO] sets the priorities now whereas programs used to be able to create that strategy envisioned for themselves. Some of that is now being driven based on [CCO] priorities and I think that might take away from some of the innovation and creativity that might have happened at a program level before”* (G25, Network, Renal)

We identified two unintended consequences in this category that are not described in the literature: (1) ‘increased perceived injustice’ and (2) ‘toxic ambition’. The literature describes ‘team and inter-professional conflict’ as an unintended consequence of PM due to workload, resource, and accountability issues [26, 27, 32, 36]. ‘Increased perceived injustice’ is similar, but in our data this occurred at the program or system level in response to social comparisons between groups affected by PM and those that are not, as this quote illustrates:

*“My colleagues in other programs sometimes feel that we are favoured or the spotlight is more on us than on them, and it creates an element of resentment, which is really not of our own doing. It’s just our requirement to report and to produce”* (G11, Network, Cancer)

‘Increased perceived injustice’ appeared to be exacerbated by ‘tunnel vision’ and ‘systemic dysfunction’.

The second novel unintended consequence in this category, ‘toxic ambition’, was uncommon and identified only among CCO representatives as they described concerns regarding continuous increases in performance targets:

*“We got lots of push back that we needed to increase [the target]. Whether that makes sense or not, I’m not sure. People are doing well and exceeding the target. Isn’t that good enough?”* (P29, CCO, Cancer, Administrator)

*“Penalizing a high performing program that isn’t getting even better? The optics are terrible…Some of them say, why do you even give us a target? Why don’t you accept that we’re doing a darn good job and move on to some other initiative? I’m sympathetic with that. I think above a certain level, we should leave them alone and stop hammering them”* (P53, CCO, Renal, Clinical Lead)

We identified very few, if any, examples of the remaining three unintended consequences in this category: ‘reduced learning and psychological safety’, ‘loss of professional ethos/morality’, and ‘bullying’.

#### Exacerbation of inequities

Exacerbation of inequities was not a theme in the data. ‘Increased resource gap’ was alluded to as a potential issue if networks fail to meet a PM requirement with funding tied to it and thus have funds clawed back, thereby further reducing their capacity to invest in improvement and achieve higher performance. The unintended consequences of ‘reduced ability to recruit necessary staff’ and ‘overcompensation’ were not identified at all.

#### Politicization of PM

We did not identify the unintended consequences of ‘political grandstanding’ and ‘political diversions’ in the data.

#### Positive unintended consequences

Participants were not explicitly asked about positive unintended consequences. Nevertheless, we identified examples of positive unintended consequences of PM on providers and organizations. Some participants described ‘improved morale’ because of PM, most often focusing on the pride associated with high performance:

*“I’ll brag if we have a top ten one because it’s good for the team to hear that, and good for those who are working hard day in and day out for the patient population…They’re proud of being high performers” (G19, Network, Cancer)*

*“The meetings happening quarterly are very helpful and give us a chance to relate to Cancer Care Ontario some of the work we’ve been doing that we’re proud of”* (G21, Network, Cancer and Renal)

PM also occasionally contributed to increased confidence and collective efficacy, as described by this network representative:

*“Our staff and our leaders said yeah, we can do something that actually improves things. You know, we don't have to be defeatist about this. We proved to ourselves that we could actually do this”* (G16, Network, Cancer, Administrator).

PM sometimes spurred ‘motivated learning and development’ beyond a reactive response to performance feedback or incentives as this quote demonstrates:

*“We’re not waiting to be told how we did. We actively look at our data on an ongoing basis and pick out where we can improve and really focus resources on that”* (G13, Network, Cancer)

‘Motivated learning and development’ often occurred together with ‘new relationships and collaborative problem-solving’. Network representatives frequently described how CCO’s PM approach spurred inter-network mentorship:

*“We really look [at the data] and reach out to other programs to see how they’re actually achieving some of their targets so we can collectively share our ideas and do better”* (G26, Network, Renal)

*“The ability to reach out to peer programs, better performing programs and gain nuggets of, wow, how have you done that, what can we pick up, what can we learn?”* (G19, Network, Cancer)

We identified one positive unintended consequence not described in the literature: ‘improved capacity planning’. A few participants described the use of information collected for PM purposes to support internal planning and external applications:

*“Although the measurement might be in order to incite improved performance…You can't create a strategic plan and get [Ministry] approval for capital initiatives in the absence of that information. So, I think it's leveraged in different ways than originally intended”* (G06, Network, Renal)

### Negative unintended consequences on patients and patient care

#### Inappropriate or sub-optimal care

Participants often questioned the extent to which PM improves quality of care, but rarely expressed concerns or shared anecdotes demonstrating that PM explicitly contributes to inappropriate or suboptimal care. In the very few examples we identified of ‘clinical decisions driven by PM’ and ‘improved documentation without improved care’, these unintended consequences seemed to stem from ‘measure fixation’ and contribute to ‘resource waste’. We did not identify any examples of ‘less continuity of care’.

#### Reduction in patient-centered care

Participants expressed more concern regarding the unintended consequences of PM on patient-centeredness than on the appropriateness of care (above). The most common unintended consequence regarding patient-centeredness was ‘compromised patient convenience’ due to PM, as this quote illustrates:

*“Even the patients are experiencing survey fatigue because we’ve got the patient experience survey that came from [CCO], but we also have our own organizational patient experience survey too…and they're not certain why they're getting surveys with similar questions”* (G23, Network, Renal)

We identified very few examples of ‘disregard for patient voice’, ‘compromised patient autonomy’, and ‘compromised patient education’, though in the few hypothetical examples shared, the three unintended consequences were intertwined:

*“If we’re supposed to be patient-centered, patient-focused, and we’re being mandated that this must be completed, we’re not truly being sensitive to the needs of the patient. Yes, this is very, very important, but, in the real world, when you’ve got that person in front of you, it may not be the time”* (P69, Network, Cancer)

*“Many of these targets involve patients. It could result, theoretically, in having pressure applied to patients, to do things that patients don’t want to do. That, to me, is a bridge too far”* (P42, CCO, Renal)

The two remaining unintended consequences in this category – ‘compromised patient engagement’ and ‘erosion of trust in care’ – were not evident in our data.

#### Exacerbation of inequities

A few participants discussed the potential for PM to inadvertently ‘increase inequities in access to high quality care’, thus ‘increasing healthcare disparities’. These participants were concerned that PM favors low-risk, mainstream patients:

*“I think that the regions trying to address performance issues for the majority of their populations, leaves out the marginalised populations – the homeless, the low-income, the ethnic populations. I think focusing on specific indicators, and moving those indicators closer to the target, can actually increase inequities in those populations because you’re focused on that indicator”* (G03, CCO, Administrators)

#### Positive unintended consequences

Participants were not explicitly asked about positive unintended consequences and we did not identify any examples of positive unintended consequences of PM on patients and patient care.

### Mitigating unintended negative consequences

CCO’s primary strategy for mitigating unintended negative consequences mirrors that in the literature: endorsement from administrative and clinical network leaders on indicators and PM interventions.

*“It’s not as though CCO comes up with these metrics and targets on their own. Provincial clinical leads and providers in our communities of practice are involved in developing them” (G15, Network, Cancer and Renal)*

*“Our stakeholder engagement is very strong. We go out to the groups, we take the initial data, we see if they have feedback and if it makes clinical sense, we rework it, if needed, then maybe eventually we get to setting a target but it’s not something we roll out right away. It’s often a year, if not more, of work and socialization” (G14, CCO, Clinical Leads, Cancer)*

In addition to involvement in indicator selection and development, ongoing dialogue between CCO and network leaders ensures that issues regarding data quality, indicator sensitivity to change, or impact on staff morale are addressed. For example, participants described the removal of two indicators from the scorecard *“at the request of the RVPs [Regional Vice Presidents]”* because they lacked responsiveness to improvement efforts and were negatively impacting staff morale (P02, CCO, Administrator).

## Discussion

Based on the results of our rapid review and qualitative study, we present a typology of 48 unintended consequences of PM in healthcare. To our knowledge, this is the most comprehensive typology of the negative and positive unintended consequences of PM and includes five unintended consequences not previously identified or well-described in the literature.

In both the rapid review and qualitative study, we found that most of the identified unintended consequences were focused on providers and organizations, not patients and patient care. Furthermore, the focus tends to be on the negative unintended consequences of PM. Very little attention has been given to identifying the positive unintended consequences of PM. Our qualitative study also did not explicitly elicit the positive unintended consequences of PM and therefore is subject to the same bias and limitation as the literature. Nevertheless, we identified ‘improved capacity planning’ as a novel positive unintended consequence of PM on organizations.

The most common unintended consequences of PM identified in the literature were measure fixation, tunnel vision, and misrepresentation or gaming, while those that were most prominent in our qualitative study were administrative burden, insensitivity, reduced morale, and systemic dysfunction. These differing results demonstrate the importance of collecting data on the unintended consequences of PM in different contexts. Results may vary due to the nature of the PM system under study, such as the extent to which PM is punitive versus supportive, or due to contextual factors, such as the extent to which healthcare organizations are subject to accountability requirements from multiple oversight bodies. Varying results may also be explained by the effects of time. PM is an evolving discipline and there has been a shift in the academic literature during the last two decades away from control- and accountability-oriented PM systems to those that are more learning- and improvement-oriented [2, 69, 70]. Paradigm shifts such as these may influence not only the nature of PM systems and therefore which unintended consequences dominate, but also how we conceptualize what is unintended versus intended. For example, while we classified “motivated learning and development” and “new relationships and collaborative problem-solving” as unintended positive consequences, given the paradigm shift just described these could be conceptualized as *intended* consequences.

In general, we did not find major differences in perceptions of unintended consequences of PM across stakeholder groups; there was significant alignment in perceptions across CCO and network representatives, cancer and renal representatives, and clinical and administrative representatives. This convergence of stakeholder views may be explained by two factors. First, CCO’s PM system involves regular feedback and discussion between CCO and the networks through quarterly performance review reports and meetings, among other methods. This ongoing dialogue means that there are ample opportunities for network representatives to bring unintended consequences to the attention of CCO representatives, and vice versa. Second, although our sample included many practicing clinicians, these individuals were in clinical leadership roles. Given their involvement in strategic decision-making, clinical leaders may be more likely than those in non-leadership positions to hold similar views as administrators. Although there were common views between cancer and renal representatives, the application of the PM system to renal care was relatively new at the time of data collection; therefore, some unintended negative consequences identified by renal representatives may reflect the early stages of implementation and change management.

We identified three key themes in our qualitative data. First, many participant comments were not about CCO’s PM approach per say, but rather about how it co-exists with the PM requirements of other oversight bodies in the healthcare system. Two of our unintended consequences – ‘systemic dysfunction’ and ‘increased perceived injustice’ – are explicitly focused on the experiences of and relationship between those subject to CCO’s PM requirements and those subject to PM requirements from other oversight bodies. Second, many of the unintended consequences identified by participants were driven by a common underlying question: “Does PM capture what matters?” This question reflects concerns with indicator selection and data availability – more so than the PM approach itself. Both of these themes can be framed as sensemaking challenges [71] in which participants struggle to make sense of what is being measured and managed, why, how, and by which oversight body. Third, we found that the unintended consequences often reinforced one another, and we included such observations throughout the results section and summarized them in Fig. 2. For example, ‘reduced morale’ was exacerbated by ‘insensitivity’, ‘increased administrative burden’ and ‘systemic dysfunction’, while ‘increased perceived injustice’ was exacerbated by ‘tunnel vision’ and ‘systemic dysfunction’. Previous studies of unintended consequences rarely examine how unintended consequences influence and mutually reinforce each other. Powell et al. [15] and Aryankhasal et al. [35] drew maps to illustrate the pathway from structures or behaviours to unintended consequences, but these maps provide minimal insight into the mutual relationships between unintended consequences.

Overall, our qualitative results suggest that the negative unintended consequences spurred by CCO’s PM system do not undermine the entire effort, but rather are side effects to be mitigated through collaborative design and implementation strategies.

### Implications for practice

This study demonstrates that even internationally leading PM systems with broad stakeholder support must grapple with unintended negative consequences of PM. Healthcare policymakers and managers can use the results of this study to inform the (re-)design and implementation of PM programs. The typology can serve as a tool to facilitate reflection and discussion on the potential risks of PM and how they may be avoided or mitigated. Our qualitative results, which provide insight into how unintended consequences manifest from multiple stakeholder perspectives and how they reinforce one another, can be similarly applied. For example, our results suggest that PM programs should be designed with consideration for how they align or conflict with PM requirements from other oversight bodies in the healthcare system. Collaborative approaches to developing PM requirements across oversight bodies will reduce conflict, confusion, and administrative burden. Our results also suggest that the unintended consequences of ‘increased administrative burden’, ‘insensitivity’, ‘systemic dysfunction’, and ‘measure fixation’ generated at least three other unintended consequences each, suggesting that particular attention should be paid to how to reduce these four core issues. Finally, we recommend pilot testing PM interventions to identify unintended consequences in real-world settings prior to full implementation [3].

### Implications for future research

Our comprehensive typology provides a common language for discourse on unintended consequences and supports systematic, comparable analyses of unintended consequences across PM regimes and healthcare systems. We recommend that researchers use and build on the typology in future studies to facilitate the accumulation of evidence on the influence of unintended consequences of PM.

The results of our rapid review and qualitative study suggest that additional research is needed on (1) the positive unintended consequences of PM, (2) how unintended consequences relate to and exacerbate each other, (3) how unintended consequences vary over time and between PM regimes and contexts and (4) mitigation strategies. Examination of these issues could help further understanding of unintended consequences and how best to mitigate those that are negative while retaining the benefits of those that are positive in diverse healthcare systems. As noted above, our qualitative results suggest that the following four unintended consequences have negative generative effects and should be prioritized in future research and practice changes: ‘increased administrative burden’, ‘insensitivity’, ‘systemic dysfunction’, and ‘measure fixation’.

Finally, in our study, we conceptualized unintended consequences from the vantage points of three core stakeholder groups - providers, organizations, and patients. We recommend researchers break these groups down further to allow for a more nuanced understanding of the question, “unintended for whom?” For example, how a PM consequence is experienced and whether it is intended or unintended may vary based on provider role, organization size or type, and patient demographics or health status.

### Study limitations

This study has limitations. First, although our literature search involved the use of several synonymous keywords and two databases reflecting the health sciences and management fields, respectively, it is possible that relevant papers were missed, particularly due to the range of possible PM interventions and differences in terminology across disciplines. Second, we did not assess the quality of studies included in the review. However, our aim was to develop a typology, not conduct a systematic review, and it is common for rapid reviews to limit sources and omit quality assessment [19]. Third, examining the unintended consequences of PM was not the research question that drove initial data collection. However, one interview question inquired about weaknesses of the PM system and another about unintended negative consequences of PM. Fourth, participants were not explicitly asked about positive unintended consequences of PM, though some were nevertheless identified in our data. Fifth, although roughly 20% of our sample consisted of practicing clinicians, these individuals held clinical leadership roles. No front-line staff in non-leadership roles were included; these individuals may have differing experiences and views of PM. Sixth, our results do not link unintended consequences with specific PM interventions; therefore, we do not know which unintended consequences are common based on type of PM intervention. Finally, since the data were collected in one Canadian province, the results may have limited generalizability. The typology, however, is rooted in both the literature and our qualitative data, and our qualitative results align with those of studies in other contexts.

## Conclusion

In this study, we undertook a rapid review and qualitative study to develop a comprehensive typology of the unintended consequences of PM in healthcare and to explore unintended consequences from multiple stakeholder perspectives. Given the increasing use of PM to drive quality and accountability in healthcare, unintended administrative and clinical implications must be considered. While unintended consequences can never be fully eliminated, we can strive to minimize those that are negative and leverage those that are positive.

## Data Availability

The data analyzed during the current study is available from the corresponding author on reasonable request.

## Abbreviations

CCO: Cancer Care Ontario
NHS: National Health Service
ORN: Ontario Renal Network
PM: Performance Management
